# Extreme dependencies and spillovers between gold and stock markets: evidence from MENA countries

**DOI:** 10.1186/s40854-023-00451-z

**Published:** 2023-02-06

**Authors:** Walid Mensi, Debasish Maitra, Refk Selmi, Xuan Vinh Vo

**Affiliations:** 1 Department of Economics and Finance, College of Economics and Political Science, Sultan Qaboos University, Muscat, Oman; 2 Institute of Business Research, University of Economics Ho Chi Minh City, Ho Chi Minh City, Vietnam; 3 Indian Institute of Management Indore, Indore, India; 4 ESC Pau Business School, Pau, France; 5 Institute of Business Research and CFVG, University of Economics Ho Chi Minh City, Ho Chi Minh City, Vietnam

**Keywords:** Copula, CoVaR, Extreme dependence, Gold, MENA markets, Risk spillovers, G14, G15

## Abstract

This study addresses whether gold exhibits the function of a hedge or safe haven as often referred to in academia. It contributes to the existing literature by (i) revisiting this question for the principal stock markets in the Middle East and North Africa (MENA) region and (ii) using the copula-quantile-on-quantile and conditional value at risk methods to detail the risks facing market participants provided with accurate information about various gold and stock market scenarios (i.e., bear, normal, bull). The results provide strong evidence of quantile dependence between gold and stock returns. Positive correlations are found between MENA gold and stock markets when both are bullish. Conversely, when stock returns are bearish, gold markets show negative correlations with MENA stock markets. The risk spillover from gold to stock markets intensified during the global financial and European crises. Given the risk spillover between gold and stock markets, investors in MENA markets should be careful when considering gold as a safe haven because its effectiveness as a hedge is not the same in all MENA stock markets. Investors and portfolio managers should rebalance their portfolio compositions under various gold and stock market conditions. Overall, such precise insights about the heterogeneous linkages and spillovers between gold and MENA stock returns provide potential input for developing effective hedging strategies and optimal portfolio allocations.

## Introduction

The gold and stock market relationship has implications for investors, traders, and portfolio managers. Due to heightened global uncertainty and its adverse impacts on financial markets, diversifying a portfolio through hedging becomes increasingly prominent. Particularly during the global financial and economic crisis of 2007–2008, the gold price witnessed a marked increase while other assets (especially stock prices) exhibited substantial losses (see Fig. 6). In this study, we revisit the role of gold as a hedge, diversifier, and safe haven asset for the principal stock markets of the Middle East and North Africa (MENA) region. To do so, we use the relatively novel copula quantile-on-quantile regression (C-QQR) method of Sim (2016) to account for extreme dependence across various quantiles. Quantile regression analysis (QRA), since its introduction by Koenker and Bassett (1978), has become a common technique for capturing the time-varying degree and structure of dependence because it involves a set of regression curves that vary across various quantiles of the conditional distribution of the dependent variable, with the quantiles detecting distinct (time-varying) phases of the dependent variable. Compared with classical linear or even nonlinear regression methods, quantile functions offer a more accurate outcome for the effects of covariates on the dependent variable. Moreover, the advantage of using QRA lies in its capability to provide information on tail dependence (i.e., upper and lower tails) in addition to the median (or normal state). Although the QRA can estimate the distinct responses of stock returns to gold returns at various points in the conditional distribution of stocks, it ignores the significant influence that the conditional distribution of gold might have on the focal relationship. This implies that by carrying out a C-QQR regression, we can provide more information on the dependence between gold and MENA stock returns. Moreover, we examine portfolio implications between gold and MENA stock markets by analyzing the risk spillover between gold and stock returns. We assess the upside and downside Value at Risk (VaR) of MENA stock markets, along with the conditional VaR (CoVaR) of portfolios with gold and stock markets, after identifying the best copula fit. Further, we evaluate the dependence between gold and the stock market across all deciles of stock market returns.

Market dependence poses challenges for investors to diversify risk when correlation across stock markets increases due to financial or macroeconomic turmoil. The series of crises, including the US subprime mortgage crisis in 2007, the global financial crisis (GFC) in 2008–2009, the European sovereign debt crisis in 2012, the great oil bust in 2014, the trade war between China and the USA, and increasing integration and recoupling among financial markets, have pushed international investors to find alternative assets that are weakly or uncorrelated with their stocks. One alternative asset group is precious metals, which have received considerable attention from investors and fund managers. One precious metal often included in portfolios to hedge stock market risk is gold. The yellow metal asset has received much attention from investors and fund managers, especially after the 2008–2009 GFC. Gold offers hedging diversification benefits for equity investors (Baur and Lucey 2010; Baur and McDermott 2010; Peng 2019), currency traders (Gürgün and Ünalmış 2014), and energy markets (Reboredo 2013a; Selmi et al. 2018). Hammoudeh et al. (2013) showed that portfolio efficiency increases more with a higher proportion of gold than with any other asset. In a recent study, Lucey and Li (2015) reported that gold performance is a better hedge for S&P 500 and US 10-year bonds, and gold provides a better safe haven investment.

An extensive body of literature on gold as an asset focusing on hedging against inflation (Salisu et al. 2019; Selmi 2022; Shahzad et al. 2019; Wang et al. 2011), protection against portfolio value loss due to a sharp fall in stock prices, and offsetting a depreciating dollar has grown (Ciner et al. 2013; Joy 2011; Qureshi et al. 2018). In theory, gold has provided flight to quality during the financial downturn and economic recession. As gold offers evidence of being decoupled from financial markets (Sunmer et al. 2010), it can be a safe haven against extreme stock market conditions, especially when market turmoil becomes evident (Baur and Lucey 2010). This paper attempts to enrich the literature on stock price crash risk (Wen et al. 2019).

The existing literature, as summarized by O'Connor et al. (2015) and Vigne et al. (2017), on the role of gold as a haven for stock markets is mostly focused on developed and major emerging markets (see, inter alia, Ming et al. 2020; Shahzad et al. 2019) and has continued to gain significantly in popularity since the financial crisis (for instance, Maghyereh et al., 2017). Some studies have focused on gold’s haven and hedging capabilities against stocks in the cases of Brazil, Russia, India, China, and South Africa (BRICS) and Asian countries (Pandey 2018; Robiyanto et al., 2019, 2022). For example, Robiyanto et al. (2020) explore the effectiveness of gold for hedging equities in the ASEAN-5 (Indonesia, Malaysia, Singapore, Thailand, and the Philippines). It is, nevertheless, scarce in MENA stock markets. Over the last decade, MENA markets have undergone considerable structural reforms and suffered from social unrest. MENA economies have experienced a series of high turbulence in the last decade due to geopolitical transitions, including the Arab Spring, the Aramco attack, the Yemen war, the Qatar–Saudi Arabia diplomatic conflict, and energy crashes (oil price plunge in 2014). Middle Eastern markets have witnessed a recent surge in portfolio inflows accounting for 20% of total portfolio investments in emerging markets (Azour and Zhu 2020).

In contrast, foreign direct investments have fallen sharply since 2008. The rise of social unrest has led to a steep decline in foreign direct investment in the MENA region (Fig. 7). It is noted that portfolio inflows to MENA markets face higher sentiment risk than global markets. For example, if the VIX doubled, portfolio investments would be halved in MENA markets (Azour and Zhu 2020). Given the higher financial market volatility in the MENA region, hedging risk with a safe haven asset becomes indispensable. Gold is a precious metal that plays an important role in the MENA region. Per capita gold consumption in the Middle East is much higher than the worldwide average. The USA, India, and China are major consuming countries, but their per capita consumption is lower than that of the Middle East region (Fig. 8). Armed with the above argument, studying the role of gold for MENA stock investors is important and timely.

This study contributes to the related literature along two dimensions. First, it extends the scant literature on the safe haven property of gold for MENA stock markets using a quantile-on-quantile approach to highlight the relationship across different market states. The literature has emphasized the gold–stock relationship, especially for developed and emerging markets; however, this relationship has not been examined for the MENA region. The economic turmoil and transitional governments experienced within the MENA region have exacerbated the uncertainty and the volatility of their financial markets, pushing investors to seek hedging assets (Chau et al. 2014). Second, our findings based on extreme risk spillover between gold and MENA stock markets shed light on whether gold can remain a good hedge or safe haven for markets when systemic risk reaches extremes. Specifically, we analyze asymmetric risk spillovers from gold to MENA stock markets by quantifying the upside and downside CoVaR of Girardi and Ergün (2013).^1^ Third, our results indicate that the responses of stock returns to gold price changes are nonhomogeneous and conditioned by respective market conditions (bear, normal, or bull). Investors and traders could use such outcomes to ensure better asset allocation in various scenarios or to build profitable speculation strategies.

The remainder of the paper is structured as follows. Sect. “Review of literature ” briefly describes the review of the literature. Sect. "Methodology" explains the methodology. Sections "Data and preliminary analysis" and “Empirical results ” discuss the data and results, respectively. Section "Conclusion" concludes and offers some policy implications.

## Review of literature

An extensive body of literature has been developed on the gold–stock relationship. Gold is considered an important financial asset that differs from other precious metals. Many studies have examined the gold and stock nexus and the possible role of gold as a hedge or safe haven.

As early as the work of Jaffe (1989), gold has been shown to help optimal portfolio diversification, where gold’s weight should not increase beyond 10% of a stock portfolio. Later, using monthly data on gold stocks, the gold index, and the S&P 500 for 1971–1988 and a market model, Chua et al. (1990) reported that gold has a low CAPM beta and gold prices do not comove with stock prices, rendering gold as a hedge against portfolio risk. A detailed investigation of gold’s diversification benefits was assessed by Hillier et al. (2006). Using the GARCH (1,1) model with daily data for gold, silver, platinum, the S&P 500 Index, and MSCI’s Europe, Australasia, and the Far East Index, they found that gold diversified stock price volatility; however, the hedging benefits of precious metals are constrained by market conditions. Gold does not offer better hedging benefits when market returns are bearish. Furthermore, the authors stated that a buy-and-hold strategy with gold invested at 9.5% in stock portfolios performed better than a switching strategy. Lucey et al. (2003), using polynomial goal programming for multimoment optimization, observed that the weights of gold become lower when skewness is considered in portfolio optimization with stocks. Mean–variance framework-based optimization requires the gold portion of the portfolio to be between 4 and 6%, whereas multimoment portfolio optimization reduces the gold investment to 2–4%.

A study by Baur and McDermott (2010) is noteworthy. This study examined the hedge and safety properties of gold against the stocks of developed markets like the G7 and emerging economies like BRICS. Employing regression between gold and stock market returns, they found strong and weak forms of the hedging and safe haven properties of gold. Gold appeared as a strong safe haven for developed markets’ stocks during the peak of the GFC. Along a similar line, Baur and Lucey (2010) regressed gold returns on stock and bond returns and reported that gold is a safe haven for stocks but not bonds. However, in the long run, gold cannot save investors from extreme negative shocks in stock markets. The results imply that investors should think of gold only during periods of extreme negative returns and sell when market volatility falls. Cohen and Qadan (2010), using a GARCH model, noted that gold shares bidirectional causality with the fear index, i.e., the US VIX, during stable market conditions. In contrast, it drives VIX changes when markets are nonstable. In contrast to existing studies, Hood and Malik (2013) extended the gold and stock market relationship by introducing the VIX as a safe haven. Using a regression model similar to that of Baur and McDermott (2010), Hood and Malik (2013) documented that gold appeared to be a hedge and weak safe haven for the US stock market. Interestingly, the VIX is a superior hedging instrument and better than gold as a safe haven.

Within a different context and with a different approach, Reboredo (2013b)^2^ employed a copula to examine the hedging and safe haven property of gold against oil prices. Data from January 2000 to September 2011 showed that gold and oil markets have a positive and significant correlation; however, there is no tail dependence. Thus, gold does not offer hedging benefits against oil. Using daily data on gold, stock, and bond prices for the US market, Lucey and Li (2014) studied the safe haven property of gold. They reported that the safe haven or hedging property of gold depends on market conditions. During some quarters, gold was a safe haven, whereas in others, it was not. In contrast to the findings of Baur and Lucey (2010), a time–frequency-based approach (wavelet) was used by Bredin et al. (2015), who observed that gold could serve as a safe haven up to one year after a market crash. Dar and Maitra (2017), using the continuous wavelet method, found that a time-varying dynamic correlation does not exist between stock and gold returns. This suggests that gold is not a good asset for portfolio diversification. A recent study by He et al. (2018) revisited gold's role in managing investors’ portfolio risk by applying CAPM to equity indices of the UK and USA and Markov switching to assess whether gold reduces portfolio risk in two distinct states. They also reported that gold is consistently a hedge, but no distinct safe haven state exists between gold and stock markets in the UK and the USA.

More recently, especially with rising uncertainty over the COVID-19 crisis, several studies have revisited the hedging and safe haven abilities of gold against stocks. The literature reveals mixed outcomes regarding the safe haven properties of this precious metal during the COVID–19 pandemic (see, inter alia, Akhtaruzzaman et al. 2021; Bouri et al. 2020; Cheema et al. 2020; Ji et al. 2020). For example, Ji et al. (2020) show that gold was a safe haven asset from December 1, 2019, to March 31, 2020, during COVID-19 using the daily returns of the MSCI equity index. Cheema et al. (2020) find that gold lost its safe haven features during the COVID-19 pandemic. Bouri et al. (2020) indicate that with growing anxiety over the pandemic, gold seems an inferior choice to Bitcoin as a safe haven. Akhtaruzzaman et al. (2021) demonstrate that dynamic conditional correlations between gold and international equity returns (S&P 500, Euro Stoxx 50, Nikkei 225, and China FTSE A50 indices) are negative during Phase I (from December 31, 2019, to March 16, 2020) of the COVID-19 pandemic, confirming the safe haven ability of gold. Nevertheless, gold lost this property for these markets during Phase II (from March 17 to April 24, 2020).

These nonhomogeneous findings offered by literature on the stock–gold nexus may be due to the sample period, the country under investigation, or the conduct of various econometric methods. Early studies are predominantly based on estimating a conventional vector error correction model (VECM). However, recent studies focus on various time horizons and market scenarios (i.e., periods of low and high volatility). For instance, Beckmann and Czudaj (2013) demonstrate that conducting a Markov switching VECM is more appropriate within this framework. Then, Beckmann et al. (2015) added that it is more effective to identify regimes without relying on a priori thresholds. They contribute to the existing literature on the focal issue by augmenting their model to a smooth transition regression by employing an exponential transition function that decomposes the regression model into two extreme regimes. One controls for periods with average stock returns, enabling an assessment of whether gold serves as a hedge for stocks. The other accounts for periods distinguished by extreme market circumstances in which the volatility of stock returns is high. Various time–frequency-based approaches (discrete wavelet decomposition and continuous wavelet) have been used to test this question (for instance, Bredin et al. 2015; Dar and Maitra 2017). The conduct of these techniques allows not solely for a discrete switching from one scenario to the other but accounts for a smooth transition between them. A discrete switching pattern seems inadequate in cases where investors with dissimilar expectations must respond rapidly, promptly, and uniformly to heterogeneous information and opportunity costs, which equates to various bands of inaction. In addition, their responses to new information might exhibit distinct delays.

Given the studies mentioned above, we find that gold is important for the diversification of stock market risk; however, a critical assessment of the gold–stock relationship is always warranted. Despite the plethora of research on the gold–stock relationship, there is a dearth of conclusive evidence. Various approaches are applied to understand the role of gold, whether it is a safe haven or merely a hedge. Nevertheless, neither the copula and quantile-based approaches nor markets other than popular emerging and developed markets have been explored. Given the importance of MENA markets in the world economy, it is pertinent to examine the relationship between the MENA region's gold and stock markets using the C-QQR approach.

We extend the existing literature in three ways. First, given the higher gold consumption in Middle East regions and the importance of this region in the world economy, we examine whether the diversification benefits of gold are available to investors in the MENA region. Second, the C-QQR method employed in the study captures the dependence between gold and stock returns at different quantiles and provides quantile correlations after estimating the copula from the marginal distributions of gold and stock returns. More precisely, C-QQR helps estimate dependence behavior under various distributions of the gold and stock markets. For example, for gold–Saudi Arabia stock returns, C-QQR allows average and tail (upper and lower) dependence to be calculated conditional on different gold and stock market return conditions: bearish, normal, and bullish. Moreover, the C-QQR approach is associated with the rapidly expanding literature on modeling dependence structure (Sim 2016). In addition, as argued by Sim (2016), most traditional methods capture correlation changes as a discrete event; hence, they fail to model dependence when markets are mildly bearish or bullish, while the C-QQR model has the advantage of analyzing market dependence under all conditions. Finally, portfolio risk analysis using the copula-based VaR and CoVaR approaches illustrates whether gold has the advantage of being a good hedge or a safe haven for stock investors in the MENA region. Such a fine analysis would help us provide crucial information to agents with respect to the MENA stock market in which they invest under various market circumstances to minimize risk and maximize returns.

## Methodology

### Copula quantile-on-quantile method

To our knowledge, the present research is the first to employ a technique that allows for asymmetry and nonlinearity to test whether MENA stock returns covary with gold returns conditional on their distinct respective market scenarios (i.e., bear, normal, or bull). More accurately, we follow Sim (2016) to combine the copula^3^ with quantile-on-quantile regression and identify the dependence structure between two variables based on the joint distributions of quantiles. C-QQR is an extension of copula regression (Bouye and Salmon, 2009) and standard quantile regression (Koenker and Hallock 2000). Unlike the quantile regression method, C-QQR tests how the quantiles of gold returns covary with MENA stock returns at each quantile.

Estimation through C-QQR involves three steps. We begin with the quantile method, whereby stock returns depend on their own lagged returns and contemporaneous gold returns. Next, we study the degree of correlation between the τ-quantile of stock returns and the φ-quantile of gold returns. Finally, the copula function is introduced to the quantile-on-quantile dependence to estimate C-QQR.

The quantile regression between stock and gold returns can be defined as

1 $$g_{t} = \gamma^{\tau } g_{t - 1} + \omega^{\tau } S_{t} + \varepsilon_{t}^{\tau }$$

We denote *S*_*t*_ and $g_{t}$ as the stock returns and gold returns, respectively, $\gamma^{\tau }$ as the coefficient of lagged gold return, and $\omega^{\tau }$ as the coefficient of contemporaneous stock returns for Eq. (1).

As prior information on the dependence between gold and stock returns is not available, $\omega^{\tau } \left( . \right)$ remains unknown. Following the strategy of Selmi et al. (2018) to estimate the correlation between the quantiles of gold and of stock returns, *ω*^*τ*^(.) is linearized by taking a first-order Taylor expansion of $\omega^{\tau } \left( . \right)$ around stock returns $S^{\varphi } .$ Thus, we obtain

2 $$\omega^{\tau } S_{t} \approx \omega^{\tau } S^{\varphi } + \omega^{{\mathop \tau \limits^{`} }} S^{\varphi } \left( {S_{t} - S^{\varphi } } \right)$$

As in the works of Sim and Zhou (2015) and Selmi et al. (2018),^4^ we redefine $\omega^{\tau } S^{\varphi }$ and $\omega^{{\mathop \tau \limits^{`} }} S^{\varphi }$ as $\omega_{0} \left( {\varphi ,\tau } \right)$ and $\omega_{1} \left( {\varphi ,\tau } \right)$, respectively. Thus, Eq. (2) can be modified as

3 $$\omega^{\tau } S_{t} \approx \omega_{0} \left( {\varphi ,\tau } \right) + \omega_{1} \left( {\varphi ,\tau } \right)\left( {S_{t} - S^{\varphi } } \right)$$

Finally, we replace Eq. (1) with Eq. (3) to obtain

4 $$g_{t} = \gamma^{\tau } g_{t - 1} + \omega_{0} \left( {\varphi ,\tau } \right) + \omega_{1} \left( {\varphi ,\tau } \right)\left( {S_{t} - S^{\varphi } } \right) + \varepsilon_{t}^{\tau }$$

Equation (4) is applied to the $\varphi$ conditional quantile of gold returns. $\omega_{0} \left( {\varphi ,\tau } \right) + \omega_{1} \left( {\varphi ,\tau } \right)\left( {S_{t} - S^{\varphi } } \right)$ captures the linkage between the $\varphi$-quantile of gold returns and the $\tau$-quantile of lagged stock returns.

Next, to model the dependence between gold and stock returns at quantiles, a copula (*h*) is introduced with a $\theta$ parameter. The copula approach has better efficiency because it identifies nonlinear associations between two series. Lastly, copula being combined with the quantile-on-quantile (Bouye and Salmon, 2009; Sim 2016) of stock and gold returns can be expressed as

5 $$g_{t} = h\left( {Fg_{t} (g_{t} ), F_{{S_{t} }} \left( {S_{t} } \right);\theta } \right)$$

$Fg_{t}$ and $F_{{S_{t} }}$ are the marginal distributions for gold and stock returns, which are combined by the copula function to form joint distributions.

In the next step, after estimating the marginal distributions of gold and stock returns, the joint distributions of gold and stock returns with the copula function (*h*) and $\theta$ parameters are estimated for quantiles of gold and stock returns:

6 $$Q_{{g_{t} }} = h\left( {Q_{{S_{t} }} ; \theta \left( {u,v} \right)} \right)$$

where *u* and *v* are the joint distributions obtained using the copula function and marginal distributions of gold and stock returns. More precisely, Eq. (6) estimates the dependence between conditional quantiles of gold returns ($\varphi_{{g_{t} }} )$ and stock return ($\tau_{{S_{t} }}$) as estimated by $\theta$ which can be summarized as $\theta \left( {F_{u}^{ - 1} \left( {\varphi_{{g_{t} }} } \right), F_{u}^{ - 1} \left( {\tau_{{S_{t} }} } \right)} \right)$.

### Gold-protection asset allocation: value at risk versus conditional value at risk

Another contribution of this research is the use of downside risk measures conditioned on different market scenarios. According to Kroner and Ng (1998)’s study, the optimal weight of gold in portfolios composed of gold and each of the MENA stocks at time *t*, respectively, is given by

7 $$w_{t}^{Gold} = \frac{{h_{t}^{stocks} - h_{t}^{Gold,stocks} }}{{h_{t}^{Gold} - 2h_{t}^{Gold, stocks} - h_{t}^{stocks} }}\;with\;w_{t}^{stocks} = \left\{ {\begin{array}{*{20}c} {0 w_{t}^{Gold} < 0 } \\ {w_{t}^{Gold} 0 \le w_{t}^{Gold} \le 1 } \\ {1 w_{t}^{Gold} > 1 } \\ \end{array} } \right.$$

where *h*_*t*_^*i*^ is the conditional volatility of *i* (where *i* corresponds to the *gold price and each MENA stock under study*), and *h*_*t*_^*Gold,stocks*^ is the conditional covariance between *gold returns and each considered MENA stock*. To determine the conditional volatility (*h*_*t*_^*i*^), a GARCH (1, 1) model is used to effectively capture the evolving volatilities of *gold and each MENA equity*.

**Fig. 1:**
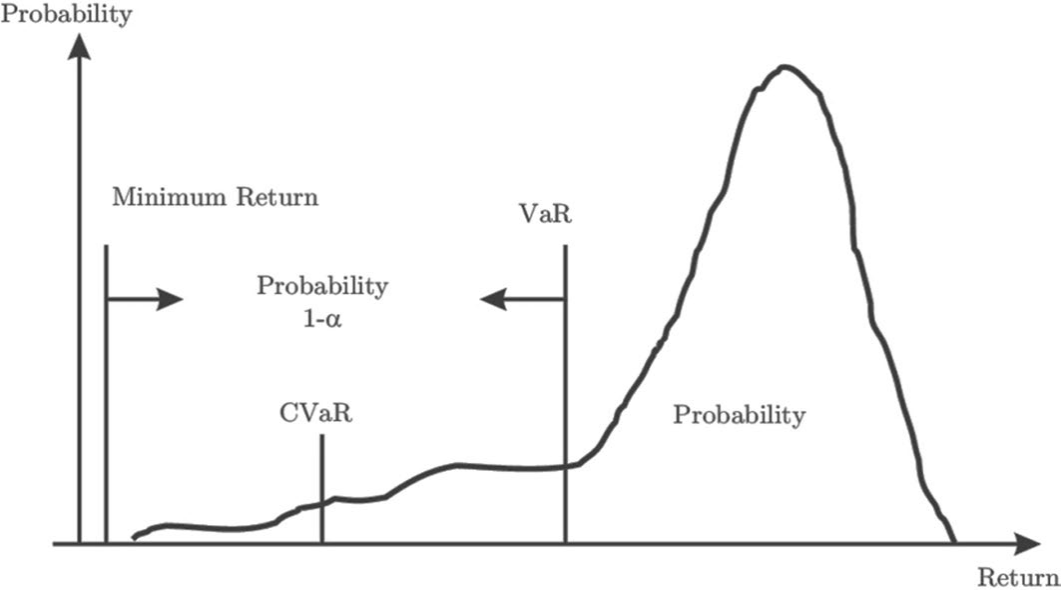
The difference between VaR and CoVaR

The optimal portfolio at time *t* results from using the information in Eq. (7) from the conditional quantile estimation of a GARCH model. Under a risk-allocation approach, it is useful to consider a broader class of processes that achieve greater flexibility by enabling the asymmetry and tail behavior of conditional return distributions. It should be noted at this stage that distributional information, such as conditional quantiles, plays a significant role in portfolio risk measurement. According to the work of Xiao and Koenker (2009), we conduct a two-step approach to the quantile regression estimation for the GARCH time series. In the first step, we conduct a quantile autoregression approximation for the GARCH model by combining information over various quantile levels. The second step consists of performing the GARCH model for the first-stage minimum distance estimation of the scale process of the focal variables. It is largely recognized that during uncertain circumstances, the loss distribution of an asset return tends to shift upward, yielding wide losses compared with normal conditions. Therefore, standard risk measures such as VaR may not succeed in effectively detecting correlation, and hence, it would be more helpful and useful to have a risk measure that captures shifts in tail correlations. Figure 1 depicts VaR and conditional value at risk (CVaR) for a given portfolio and the confidence level *α*. Let *α* ∈ (0,1) be the confidence level. The VaR at a 100α% confidence level is the expected return of a given portfolio such that for 100α% of cases, the return will not surpass the VaR. However, the CVaR at a 100α% confidence level is the anticipated return of a portfolio in the worst 100(1 − *α*)% of cases, allowing 100(1 − *α*)% of the output to not overreach the VaR.

The VaR provides the maximum loss in portfolio value for a specific period and confidence level. At time *t*, the VaR for a portfolio with return *R*_*t*_ is given by the *p*^th^ percentile of the return distribution:

8 $$\Pr (R_{t} \le VaR_{t} \left| {\psi_{t - 1} ) = } \right.p$$

where *y*_*t − 1*_ is the information provided at *t* − 1; the VaR is expressed as follows:

9 $$VaR_{t} (p) = \mu_{t} +^{t - 1}_{v} (p)\sqrt {h_{t} }$$

where *m*_*t*_ and $\sqrt {h_{t} }$ are the conditional mean and standard deviation for asset returns, and *t*_*v*_^−1^(*p*) denotes the *p*^th^ quantile of the student’s t-distribution with *v* degrees of freedom.

The CVaR corresponds to the q-percent VaR value for asset *j* when asset *i* is at its *q*-percent VaR value. Overall, the CoVaR of a particular portfolio of assets consists of the VaR of this portfolio conditioned upon distinct market conditions.

## Data and preliminary analysis

**Fig. 2:**
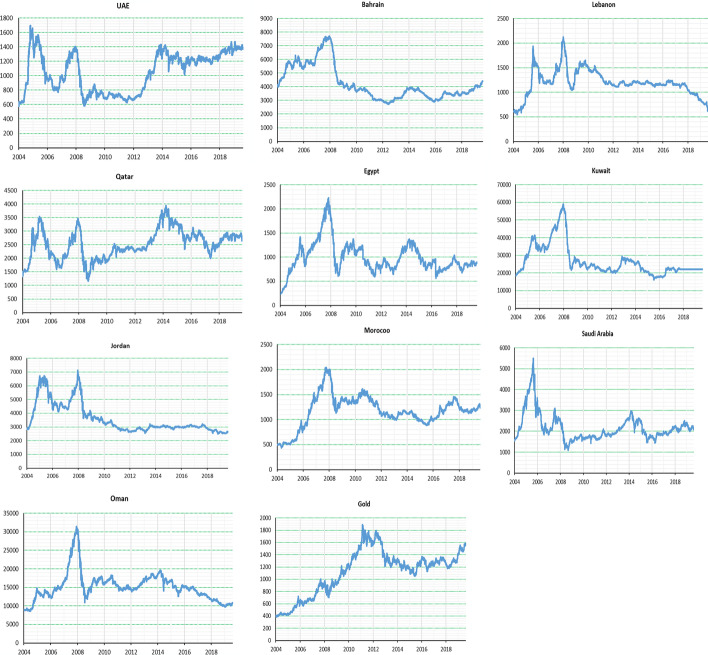
Time evolution of MENA stock indices and gold prices ($/ounce).* Notes* The above figures show the dynamics of stock market indices of the MENA region and gold prices. The X-axis (Y-axis) indicates time and index or price level ($/ounce).

We consider daily closing prices of gold and MENA stock market indices, including in the UAE, Bahrain, Lebanon, Qatar, Egypt, Jordan, Kuwait, Morocco, Oman, and Saudi Arabia. We also consider international gold prices ($ per ounce). The sample period ranges from July 2004 to February 2020. The data are sourced from the Thomson Reuters database (https://www.reuters.com/markets/quote/XAU=X/) or https://goldprice.org/gold-price.html. Figure 2 exhibits the time evolution of gold and MENA stock prices. As we can see, all stock markets witnessed upward movement from 2004 until 2008. In 2008, a significant decline was seen owing to the 2008 GFC. In addition, a downward trend can be seen during 2016–2018 for the stock markets of Lebanon, Qatar, Jordan, and Oman. In contrast, gold prices continuously rose from 2004 until 2011, after which they fell before starting to soar in 2016.

Table 1 summarizes the summary statistics of MENA stock market returns and gold price returns. The average returns are positive for all stock markets except Jordan, Kuwait, and Oman. Morocco exhibits the highest returns, followed by Qatar's stock markets and the UAE. Among MENA markets, Egypt shows the highest volatility (1.577), followed by the Qatar and Saudi Arabian stock markets. Gold shows the highest daily average returns (0.033 percent), with volatility similar to Egypt, Qatar, and Saudi Arabia. Stock and gold price returns are negatively skewed, indicating asymmetry. The higher kurtosis value suggests fat tails of stock market returns. All return series digress from normal distributions, as shown by the Jarque–Bera statistic. The last row of Table 1 indicates that gold is negatively (positively) correlated with the stock markets of UAE, Egypt, Jordan, Lebanon, and Saudi Arabia (Bahrain, Kuwait, Morocco, Oman, and Qatar).

**Table 1:** Descriptive statistics

	UAE	Bahrain	Egypt	Jordan	Kuwait	Lebanon	Morocco	Oman	Qatar	Saudi Arabia	Gold
Mean (%)	0.015	0.0015	0.0117	− 0.0100	− 0.0019	0.0005	0.0231	− 0.0066	0.0198	0.0064	0.0338
Max (%)	8.290	3.6131	10.988	4.6088	3.4196	8.4902	4.9922	8.0388	9.4219	8.7496	8.6249
Min (%)	− 8.679	− 4.9279	− 40.589	− 4.5256	− 6.2416	− 10.687	− 6.8181	− 8.6989	− 9.1569	− 10.519	− 9.8205
S. Dev. (%)	0.9712	0.4936	1.5777	0.7025	0.5765	0.9392	0.8819	0.8143	1.1685	1.1163	1.1115
Skewness	− 0.084	− 0.4184	− 4.9926	− 0.5087	− 1.2535	− 0.5442	− 0.1290	− 1.2916	− 0.3575	− 1.1079	− 0.3513
Kurtosis	17.29	12.81	118.89	11.40	13.78	32.01	7.90	29.75	13.65	20.31	9.11
J-B	51,578.70	16,466.46	2,297,435	12,171.36	20,814.91	143,154.5	4089.694	122,697.2	19,374.69	51,759.94	6433.869
Probability	0.00	0.00	0.00	0.00	0.00	0.00	0.00	0.00	0.00	0.00	0.00
Correlation	− 0.0292	0.0093	− 0.0135	− 0.0022	0.0230	− 0.0314	0.1917	0.0064	0.0127	− 0.0026	
ADF	− 40.16***	− 67.80***	− 40.05***	− 40.31***	− 23.11***	− 40.59***	− 64.85***	− 40.01***	− 65.35***	− 71.74***	− 63.94***
PP	-68.01***	-69.18***	-68.31***	-65.93***	-75.03***	-68.28***	-64.28***	-62.71***	-65.37***	-71.18***	-63.94***

This table presents the descriptive statistics of the price returns of stock markets of MENA region (UAE, Bahrain, Egypt, Jordan, Kuwait, Lebanon, Morocco, Oman, Qatar, Saudi Arabia) and the gold market. S.Dev implies standard deviation. J-B refers to Jarque Bera test. *** suggests significant at 1% level of significance

## Empirical results

### C-QQR analysis

**Fig. 3:**
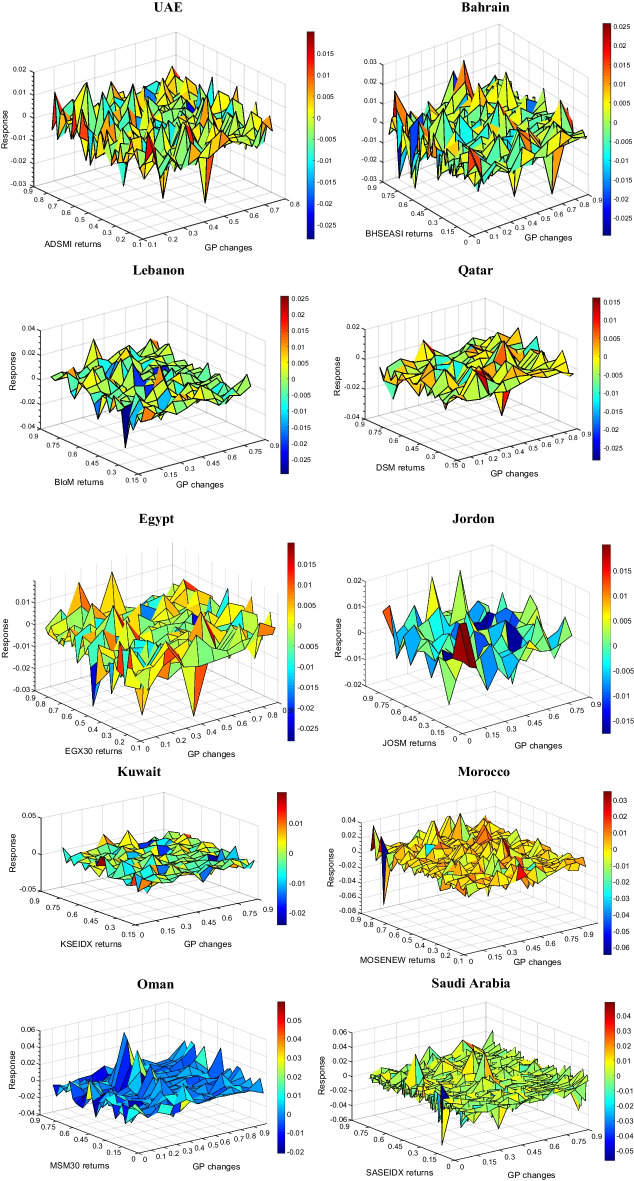
Copula quantile-on-quantile (C-QQR) between MENA gold and stock markets

C-QQR estimates the correlations between MENA gold and stock returns at various quantiles ranging from 0.1 to 0.9 (Fig. 3). Thus, we identify the correlations between the 10^th^ percentiles of gold returns with the 10th, 20th, 30th…90th percentile returns of one particular stock market. This process helps us capture the dependence of gold and the stock market at the 10th, 20th, and 30th percentiles (70th, 80th, and 90th) when the market condition is bearish and falling downward (optimistic and bullish). The steady state is denoted by the 40th, 50th, and 60th quantiles. Summing up, the quantiles reflect the degrees of bearishness, normalcy, or bullishness for the respective markets.

When stock markets are in a bullish phase, we note positive correlations between gold and stock returns when gold returns are also in a bullish state; however, the correlations are negative between stock and gold returns in Saudi Arabia when the gold market is in a bullish phase (Fig. 3). However, the positive correlations are between 0.01 and 0.02. The results suggest that gold is a good hedge to the stock market during bull market conditions. UAE also shows negative correlations between the stock market and gold markets when the former is bullish and the latter is bearish. Conversely, when stock returns are bearish, gold markets show negative correlations with all stock markets of the MENA region. The negative correlations increase when gold markets are between a steady and bearish state. The correlations range between − 0.02 and − 0.04; however, we see higher negative correlations between the stock returns of UAE, Lebanon, and Saudi Arabia and gold returns when both markets are in a bearish state. The findings suggest that when stock markets show a downward trend, gold prices show negative correlations, given that the gold prices are already in a bearish state. However, similar negative correlations exist when stock markets are bullish. This indicates that gold can be a good hedge for MENA stock markets. This is due to the fact of flight to quality and flight-to-safety phenomena of investors. The existing literature corroborates the results (Baur and McDermott 2010; Bredin et al. 2015; Ciner et al. 2013; Mensi et al. 2015; Raza et al. 2016) that gold is a good hedge, if not a safe haven, helping to diversify stock market risk.

To show the dependence structure more clearly, we report the dependence between gold and stock market returns of the UAE in Table 2.^5^ The lower or higher quantiles of gold and UAE stock market returns signify the extreme conditions of variations, and the medium quantiles of gold and stock market returns indicate little or no change. For the gold and UAE stock market returns, the overall dependence is increasing negatively, showing that dependence is larger with the upper quantiles of UAE stock market returns (between 0.05 and 0.95) and lower quantiles of gold returns (0.05 and 0.1). We also note a similar trend with the upper quantiles of gold and lower quantiles of UAE stock markets. However, the negative dependence is also seen at lower to medium quantiles of stock and gold returns. Similar to Fig. 2, the findings suggest that when stock markets show a downward trend, gold prices show negative correlations, given that the gold prices are already in a bearish state.

**Table 2:**
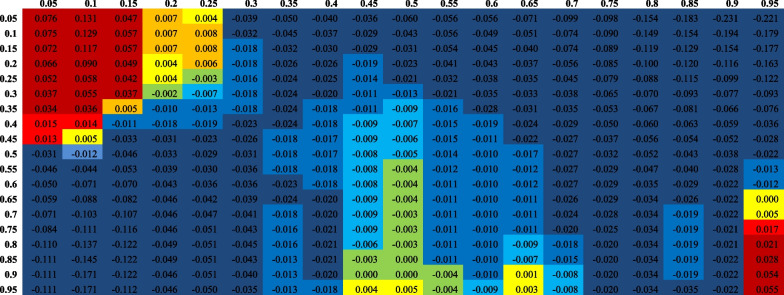
Quantile-on-quantile dependence between gold and UAE stock returns

The table reports the quantile-on-quantile dependence between gold and stock market returns of UAE. The left (top) column (row) indicates gold (UAE stock market) returns across quantiles 0.05 to 0.95. Lower or higher quantiles of gold and UAE stock market returns signify the extreme conditions of variations, and the medium quantiles of gold and stock market returns indicate little or no change

To ascertain the robustness of our findings, we assess how different econometric specifications may change our estimates. To do so, we compare the C-QQR findings with those of the QRA. The C-QQR consists of disentangling the QRA estimates so that they are specific estimates of the parameters for the different quantiles of the independent time series. Indeed, the QRA cannot properly describe the entire dependence between gold returns and each of MENA stock returns. While the QRA appears able to estimate the various responses of the MENA stock returns to gold returns at various points of the conditional distribution of the different MENA stocks under study, it ignores that the gold market state might also have an impact on the focal dependence. In the following, we compare the QRA estimates with the τ-averaged C-QQR parameters. But before starting this investigation, it must be stressed that the C-QQR regresses the θ-quantile of MENA stock returns on the τ-quantile of gold returns (double indexing, i.e., θ and τ), whereas QR regresses the θ-quantile of MENA stock returns on extreme gold returns (solely indexed by θ). This implies that using C-QQR would provide more information on the dependence structure between the gold and MENA stock returns.

When we construct the parameters from the C-QQR model that are indexed by θ, the estimated C-QQR parameters are displayed by averaging over τ. Consequently, the effects of the gold returns on the distribution of the MENA stock returns are denoted by $\hat{\gamma }_{1} (\theta )$:

10 $$\hat{\gamma }_{1} (\theta ) = {\raise0.7ex\hbox{$1$} \!\mathord{\left/ {\vphantom {1 s}}\right.\kern-0pt} \!\lower0.7ex\hbox{$s$}}\sum\limits_{{}} {}_{\tau } \hat{\beta }_{1} (\theta ,\tau )$$

where *s* = *20* is the number of quantiles *τ* = *0.05, 0.1, …, 1*.

**Fig. 4:**
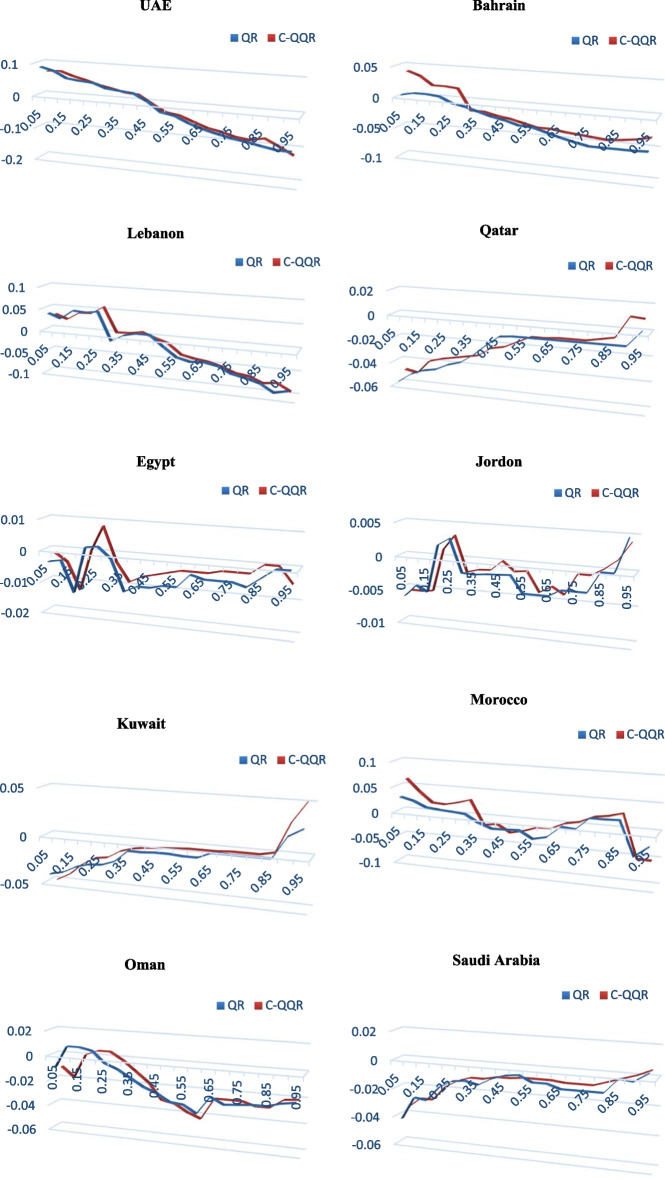
Copula quantile-on-quantile (C-QQR) versus quantile regression analysis (QRA) between MENA gold and stock markets

Figure 4 depicts the trajectory of the QRA and the averaged C-QQR estimates of the slope coefficient that measures the dependence structure of gold returns and each of MENA stock returns. Figure 4 provides a simple validation of the C-QQR methodology by revealing that the features of the quantile regression model can be recovered by summarizing the detailed information incorporated in the C-QQR estimates. However, it must be pointed out at this stage that C-QQR offers more complete information about the linkage between MENA stock returns and gold returns than the QRA, as the latter does not control for the possibility that various gold market states may also significantly affect the interdependency of MENA stock and gold returns.

### Implications on portfolio risk management

As a final step of our assessment, we investigate the importance of our findings from an economic perspective by underscoring the effectiveness of our outcomes in the context of asset management. Specifically, we analyze the risk spillover between gold and stock returns. To show that our results are valid for various market circumstances, we consider different hedge and safe haven compositions while focusing on various scenarios.

We begin with the identification process of the best copula fit to estimate the VaR and CoVaR (Table 5).^6^ Table 5 in the appendix reveals that gold returns show an asymmetric time-variant relationship with four stock markets—Bahrain, Lebanon, Qatar, and Jordan—whereas gold shares lower tail dependence with the stock returns of Egypt, Kuwait, Morocco, Oman, and Saudi Arabia.^7^

**Fig. 5:**
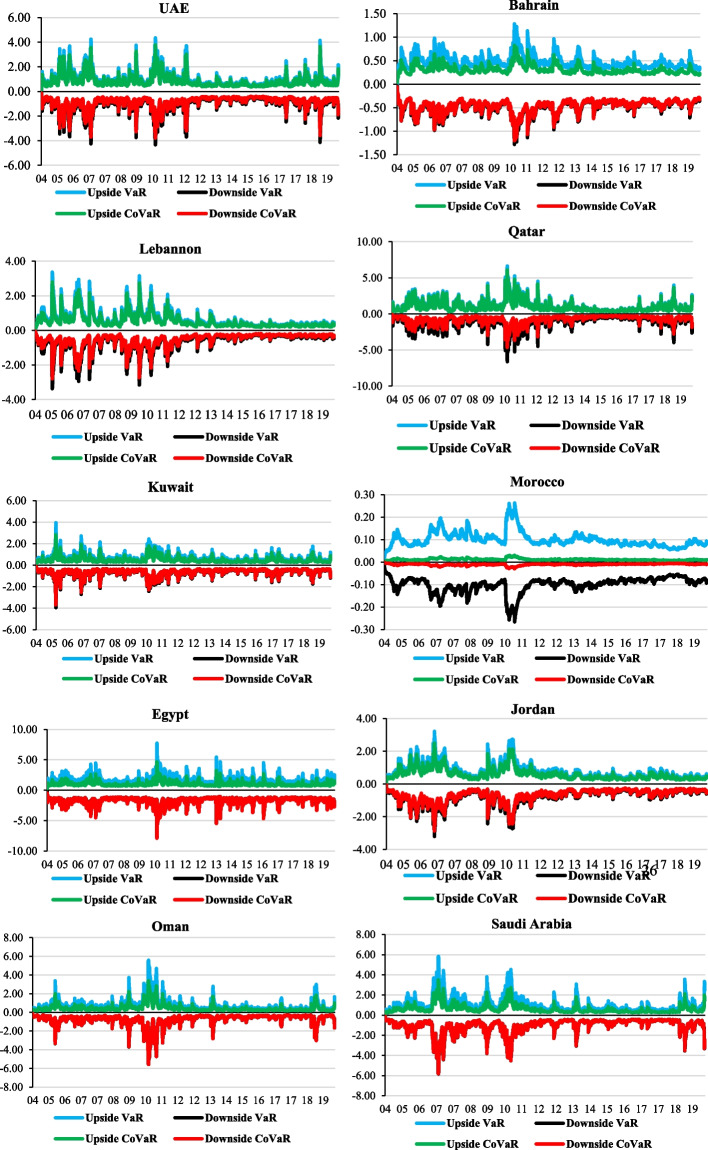
Upside and downside Value at Risk and Conditional Value at Risk. The above figure exhibits the upside/downside VaR of stock markets and CoVaR from gold to stock markets

**Fig. 6:**
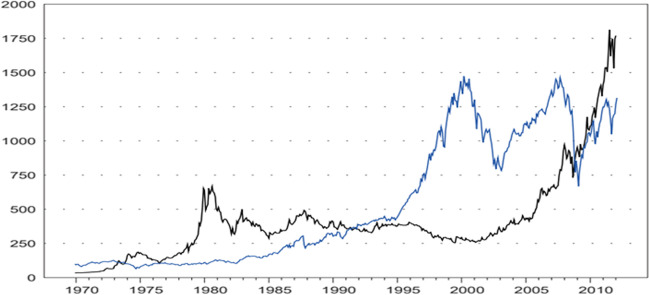
Gold prices (black line) and US stock prices (blue line) both in US dollar

**Fig. 7:**
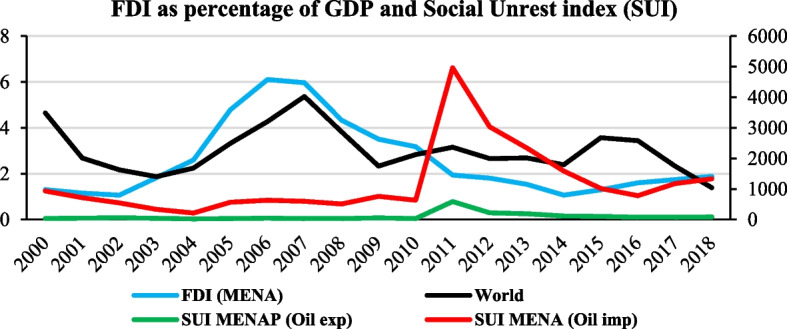
FDI and Social Unrest in MENA region

**Fig. 8:**
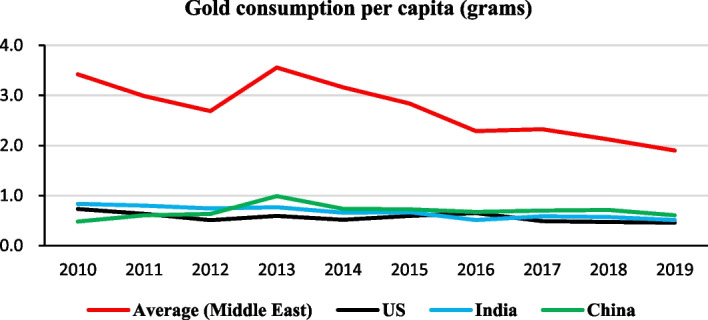
Per capita gold consumption-Middle East, the US, India, and China

Further, we assess the upside and downside VaR of MENA stock markets, along with the CoVaR of portfolios of gold and stock markets, after identifying the best copula fit. Figure 5 depicts that the VaR of stock market returns of the MENA region is higher than for gold returns, suggesting that stock market returns are riskier than the gold market when both the markets are bullish (upside) and bearish (downside). However, in Egypt and Saudi Arabia, we note the downside risk of gold returns is higher than or similar to the stock market returns when the stock market is bearish. This finding corroborates the results of C-QQR of the stock market and gold market returns. We observe that VaR and CoVaR intensified during 2007–2008 and 2010–2012, overlapping with the 2008 GFC and the eurozone debt crisis, respectively. Interestingly, oil-exporting countries like UAE, Qatar, Oman, and Saudi Arabia also show peaks of risks during 2018–2019, which is owing to the worldwide oil price crash.

In continuation of Fig. 5, we also summarize VaR and CoVaR statistics in Table 3. We find that the average upside and downside CoVaRs are lower than the upside and downside VaRs of stock markets, except Egypt. However, for UAE, Morocco, Oman, and Saudi Arabia, the downside CoVaR is similar to the downside VaR, indicating gold has significant spillover effects. It may not be a safe haven for these stock markets. This could be because the per capita gold consumptions of UAE and Saudi Arabia are very high, and they do not use gold as a flight-to-safety asset. For other MENA markets, for example, Bahrain, Lebanon, Qatar, Jordan, and Kuwait, gold reduces the risk of the stock markets very negligibly. This suggests the unique and distinctive property of gold and its usefulness as a financial asset (Batten et al. 2010) for these MENA markets can be considered only as a hedge, not as a safe haven. Please recall that an asset is perceived as a safe haven if it is uncorrelated or negatively correlated with another asset or portfolio in times of market stress or turmoil.

**Table 3:** Summary value at risk and conditional value at risk statistics for stock markets in the MENA region

Market	Upside VaR	Downside VaR	Upside CoVaR	Downside CoVaR
UAE	1.033 (0.70)	− 1.032 (0.70)	0.619 (0.42)	− 1.03 (0.70)
Bahrain	0.484 (0.146)			
	− 0.486 (0.146)	0.313 (0.095)	− 0.461 (0.139)	
Lebanon	0.651 (0.481)	− 0.651 (0.481)	0.542 (0.397)	− 0.541 (0.397)
Qatar	1.077 (0.760)	− 1.076 (0.760)	0.988 (0.697)	− 0.755 (0.533)
Egypt	1.702 (0.594)	− 1.700 (0.594)	1.025 (0.358)	− 1.731 (0.608)
Jordan	0.766 (0.450)	− 0.766 (0.450)	0.596 (0.351)	− 0.684 (0.403)
Kuwait	0.670 (0.340)	− 0.669 (0.340)	0.484 (0.247)	− 0.626 (0.318)
Morocco	0.100 (0.033)	− 0.100 (0.033)	0.011 (0.003)	− 0.010 (0.003)
Oman	0.745 (0.603)	− 0.745 (0.603)	0.446 (0.362)	− 0.741 (0.601)
Saudi Arabia	1.033 (0.703)	− 1.032 (0.703)	0.619 (0.420)	− 1.030 (0.700)

The table reports the mean and standard deviations (in the first bracket) of VaRs of the stock markets and CoVaRs of portfolios with stock and gold markets. The values in bold suggest higher risk and risk spillover

### Dependence at deciles of stock market return

Furthermore, we also examine gold and stock market dependence of the MENA region based on dependence dynamics across different deciles of stock market returns. The results in Table 4 reveal that gold has a negative correlation with the stock markets of the UAE, Lebanon, Egypt, Jordan, and Saudi Arabia. Interestingly, we note a constant sign and degree of correlation across all deciles of stock market returns in the UAE, Lebanon, Egypt, Jordan, and Saudi Arabia. The findings suggest that gold is a better hedge for these stock markets compared with the stock markets of Bahrain, Qatar, Kuwait, Morocco, and Oman, where gold has a positive correlation with stock returns across all deciles. The findings suggest that gold is a good hedge only for a few MENA markets; however, the role of gold as a safe haven for MENA markets cannot be confirmed.

**Table 4:** Conditional correlations and deciles

Decile Equity Returns	Conditional correlations with gold									
	UAE	Bahrain	Lebanon	Qatar	Egypt	Jordan	Kuwait	Morocco	Oman	Saudi
Arabia										
*Lowest*	− 0.024 (0.017)	0.016 (0.015)	− 0.027 (0.015)	0.016 (0.021)	− 0.002 (0.016)	− 0.022 (0.019)	0.012 (0.019)	0.188 (0.025)	0.014 (0.023)	− 0.018 (0.015)
2^nd^	− 0.024 (0.016)	0.013 (0.012)	− 0.03 (0.015)	0.01 (0.016)	− 0.002 (0.016)	− 0.024 (0.016)	0.014 (0.021)	0.186 (0.024)	0.011 (0.022)	− 0.016 (0.015)
3^rd^	− 0.023 (0.014)	0.012 (0.014)	− 0.03 (0.016)	0.011 (0.016)	− 0.001 (0.017)	− 0.026 (0.017)	0.013 (0.02)	0.186 (0.022)	0.01 (0.022)	− 0.016 (0.015)
4^th^	− 0.022 (0.015)	0.012 (0.013)	− 0.03 (0.015)	0.01 (0.015)	− 0.001 (0.014)	− 0.029 (0.015)	0.002 (0.016)	0.184 (0.023)	0.014 (0.02)	− 0.015 (0.013)
5^th^	− 0.027 (0.015)	0.015 (0.016)	− 0.031 (0.014)	0.018 (0.018)	0.005 (0.016)	− 0.015 (0.019)	0.003 (0.023)	0.186 (0.022)	0.016 (0.018)	− 0.013 (0.018)
6^th^	− 0.022 (0.018)	0.014 (0.013)	− 0.031 (0.015)	0.009 (0.017)	− 0.01 (0.015)	− 0.031 (0.015)	0.013 (0.016)	0.185 (0.02)	− 0.002 (0.018)	− 0.019 (0.013)
7^th^	− 0.021 (0.015)	0.011 (0.01)	− 0.031 (0.014)	0.015 (0.015)	− 0.002 (0.016)	− 0.025 (0.016)	0.019 (0.021)	0.184 (0.023)	0.016 (0.023)	− 0.019 (0.015)
8^th^	− 0.023 (0.015)	0.012 (0.012)	− 0.031 (0.015)	0.013 (0.017)	− 0.001 (0.018)	− 0.025 (0.017)	0.012 (0.021)	0.184 (0.022)	0.011 (0.021)	− 0.016 (0.015)
9^th^	− 0.024 (0.016)	0.013 (0.013)	− 0.031 (0.016)	0.011 (0.016)	− 0.002 (0.018)	− 0.026 (0.016)	0.011 (0.021)	0.184 (0.022)	0.008 (0.019)	− 0.015 (0.016)
Highest	− 0.024 (0.016)	0.014 (0.013)	− 0.027 (0.015)	0.011 (0.019)	− 0.001 (0.017)	− 0.024 (0.019)	0.01 (0.021)	0.185 (0.023)	0.014 (0.021)	− 0.016 (0.015)

This table shows mean dependence of gold returns for various deciles of equity returns of MENA region. The values in parentheses are asymptotic standard deviations for mean dynamic conditional correlations

To sum up, our main results show strong evidence of positive correlations between gold and stock returns when both markets are in a bullish state. However, gold shows negative correlations when stock markets are in a bearish state, provided that the gold market remains between a steady and bearish state. The results suggest gold may be a good hedge but cannot be a safe haven for stock returns in MENA markets; however, this is true for all MENA markets alike. The findings also indicate that MENA countries with higher per capita gold consumption, such as UAE and Saudi Arabia, do not significantly improve risk hedging after including gold in their portfolios. Gold’s hedging ability is stronger for UAE and Saudi Arabia than other MENA markets (Bahrain, Lebanon, Qatar, Jordan, and Kuwait), as there is a negligible improvement in downside risk when gold is considered in portfolios with stocks. The results offer a better way to strategize portfolios to avoid losses during extreme events.

## Conclusion

In this study, we extend prior research by adopting a novel quantile-based approach, namely C-QQR, to address whether gold can be considered a hedge or safe haven with regard to MENA stocks under various market scenarios. Most previous studies overlook nonlinear and tail dependence despite their paramount prominence in the sense that first, linear correlation measures, in many cases, may disregard potential dependence between two series and result in a nonrelation result. Second, prior research assesses the safe haven capabilities of gold against stocks by considering a threshold or time-varying model; nevertheless, the correlation coefficient is insufficient to appropriately depict the dependence structure based on Embrechts et al.’s (2003) study, particularly when the joint distribution of gold and stocks is far from elliptical. Additionally, the marginal effects captured by the threshold approach do not completely control for joint extreme market fluctuations. Thus, we conduct C-QQR to address gold as a hedge or safe haven for MENA stocks, as it offers precise information on the dependence structure and is a more flexible modeling tool than parametric bivariate distributions. This new technique provides a measure of average dependence and a gage of upper and lower tail dependence, conditional on three gold and stock market conditions (i.e., bear, normal, or bull). This implies that quantile-on-quantile regression can offer more information on the dependence between gold and MENA stock markets to minimize risk and maximize returns. Additionally, we examine the average and extreme upside and downside systemic risk spillovers between gold and stock markets using the best-fit copula structure to look at portfolio risk management in a new way.

Our results deeply underscore the importance of disentangling gold returns and stock returns into their various investment horizons (Beckmann and Czudaj 2013; Beckmann et al. 2015) and, more interestingly, the role of the conduct of estimation methods controlling for information from quantiles for both gold returns and stock returns. More specifically, our findings provide strong evidence of quantile dependence between gold and stock returns. There are positive correlations between MENA gold and stock markets when both markets are bullish. Conversely, when stock returns are bearish, gold markets show negative correlations with the MENA region’s stock markets. Negative correlations increase when gold markets are between steady and bearish states. However, decile-wise results suggest that gold cannot be a safe haven for MENA stock market returns. Our findings from systemic risk spillovers at extremes suggest that UAE, Egypt, Morocco, Oman, and Saudi Arabia do not significantly improve risk hedging after including gold in their portfolios. In contrast, other MENA regions (Bahrain, Lebanon, Qatar, Jordan, and Kuwait) show negligible improvement in downside risk when gold is considered in stock portfolios.

The novelty of this study relies on deducing that (a) the ability of gold to act as a hedge or safe haven with regard to MENA stocks is conditional on varying gold market states and MENA stock market conditions, and (b) the relationship between gold and MENA stocks is nonlinear, probably owing to the behaviors of gold traders and MENA investors, the interplay of supply and demand in the gold market, price fluctuations of other assets, and implemented monetary policies. Given the risk spillover between gold and stock markets, investors in MENA markets should be careful when considering gold as a safe haven, as its hedging strength is not the same in all MENA stock markets. Similarly, policymakers should intervene when stock markets fall sharply to restore financial stability by lowering systemic risk.

Our results are quite timely and useful for individual and institutional investors. The global financial markets continue to be persistently rocked by unpredictable and extremely destabilizing events, and MENA stock markets are no exception. In today’s uncertain environment, having accurate insights about gold price dynamics and comovements with stocks under various scenarios becomes fundamental in designing appropriate risk management strategies. When situations of heightened uncertainty or anxiety arise, an effective defense is being well informed. Throughout this analysis, we detail the risks facing MENA market participants while offering precise information about several scenarios, particularly how to deal with worst-case situations.

Some limits of our study should be underscored. Recently, the Russian/Ukrainian war has been an unusual event that has sparked an uptick in demand for gold as a safe haven asset, as investors adopt a risk-off sentiment in distressing times. In just a few days, Russia’s invasion of Ukraine yielded a series of economic maneuvers that rapidly transformed how countries raise money, where they buy raw materials, and with whom they do business. Therefore, more research is required to better understand the increasing uncertainties over geopolitical tensions and their implications for MENA stock markets and gold’s safe haven property. Another point related to using stock indices is that dissimilarities in the compositions of indices across countries could significantly affect our findings. For example, Kuwait, Saudi Arabia, Qatar, and UAE have more natural resource companies in their indices than many other countries. Thus, we would expect to see varying dependence between gold and the indices in these countries and gold and the indices from countries with less resource exposure. One way to address this concern in future research could be to conduct the same analysis but for industry-specific indices from each country to more clearly identify the role of gold. Finally, the world has witnessed an unprecedented event with the emergence of COVID-19, which has generated significant financial and psychological uncertainty in various industries and economies (Khou et al., 2021). COVID-19 has yielded relevant questions about the most appropriate hedging functionality of various assets against pandemic-related risks (Corbet et al. 2022). Given this, the period under study could be extended to account for periods following COVID-19 and the war in Ukraine and their impact on gold’s hedging effectiveness against GCC equities. This extension of the work is beyond the scope of the present research.

## Data Availability

The datasets used and analyzed during the current study are available from the corresponding author on reasonable request.

## Appendix

See Figs. 6, 7 and 8 and Table

**Table 5:** Time varying copula dependencies between Gold and MENA stock returns

	UAE	Bahrain	Lebanon	Qatar	Egypt	Jordan	Kuwait	Morocco	Oman	Saudi Arabia
1. Normal										
ὼ	− 0.0129***	− 0.0097***	− 0.0004***	− 0.0050***	0.0100***	0.0267***	− 0.0167***	− 0.0062***	− 0.0288***	0.0151***
	(0.0519)	(0.0477)	(0.0027)	(0.0172)	(0.0361)	(0.0506)	(0.0580)	(0.0646)	(0.0489)	(0.0216)
Α	0.0744***	0.1405***	0.0298***	− 0.0175***	0.0136***	− 0.1812***	0.1552***	0.0089***	0.1113***	0.0300***
	(0.1374)	(0.1370)	(0.0220)	(0.0651)	(0.0473)	(0.1463)	(0.1500)	(0.0667)	(0.1446)	(0.0474)
Β	− 1.2705***	− 1.0026***	1.8333***	1.2815***	1.5478***	− 1.2041***	− 1.7655***	1.4258***	− 0.9030***	1.3999***
	(1.6929)	(1.1338)	(0.1450)	(2.4968)	(1.6280)	(0.9040)	(0.3499)	(5.5887)	(1.8628)	(0.7513)
AIC	− 0.3578	− **1.1468**	− **4.3074**	− **0.3989**	− 2.3654	− **1.8458**	− 1.1473	− 0.5528	− 1.0655	− 3.6353
2. Clayton										
*Ψ*_0_	0.0004	− 0.0295***	0.2276***	0.0003	− 0.3487	− 0.0375***	− 0.2167***	0.0008	0.0011	0.2262***
	(0.9263)	(0.1599)	(0.0942)	(2.4083)	(13,635.2541)	(0.1551)	(0.0180)	(0.8112)	(0.3720)	(2.2909)
Ψ_1_	− 2.2050***	2.2990***	1.2155***	− 1.8390***	− 2.1367	− 0.7764***	− 2.0439***	− 1.8541***	− 1.6525***	1.7016***
	(3.0071)	(0.2748)	(0.2254)	(27.4406)	(906.3707)	(0.3138)	(0.1017)	(0.7720)	(1.4331)	(0.9396)
Ψ_2_	− 0.0005	0.1906***	− 0.5370***	− 0.0002	1.2314	0.3049***	0.6591***	− 0.0018	− 0.0026	− 0.4038**
	(2.9567)	(0.1667)	(0.3633)	(2.3608)	(2309.4563)	(0.6321)	(0.0776)	(3.2988)	(5.3837)	(12.8626)
AIC	0.0162	− 0.0275	− 0.2052	0.0179	− 1.4577	− 0.1066	− 0.0688	0.0221	0.0246	− 3.9744
3. Rotated clayton										
ὼ	− 0.1590***	0.0010***	− 0.4331***	0.2280***	0.0435***	0.3762	0.5092***	0.4016***	0.5687***	0.6538***
	(0.0920)	(0.0236)	(0.2877)	(1.9164)	(0.6196)	(29.2864)	(0.1439)	(0.2494)	(0.0378)	(0.0370)
Α	− 3.6261***	− 2.6439***	1.0282***	− 1.4039***	− 0.5947***	− 2.6577***	− 1.2069***	− 3.3608***	− 0.8150***	− 2.9407***
	(2.2991)	(0.1014)	(0.3208)	(0.7755)	(0.7205)	(13.9606)	(0.0836)	(3.5003)	(0.0692)	(0.1309)
Β	0.9571***	− 0.0017***	1.3500***	− 0.4450***	0.4718***	− 0.8237***	− 1.2953***	− 0.9125***	− 1.6003***	− 1.3674***
	(0.3399)	(0.0247)	(0.6709)	(2.5652)	(1.7148)	(15.6611)	(0.4530)	(0.5690)	(0.0867)	(0.1571)
AIC	**− 1.4283**	0.0061	− 3.4085	− 0.2153	− 4.2741	0.5554	**− 1.9038**	**− 0.8646**	**− 4.4927**	**− 5.3361**
4. Gumbel										
ὼ_u_	− 0.0004	0.0003	2.1889***	0.0000	3.5819***	− 0.1043***	2.2448***	0.0370	1.6511***	2.0752***
	(7.8567)	(9.8943)	(0.8930)	(3.6213)	(0.5818)	(2.3928)	(1.5093)	(5.2778)	(0.4103)	(7.4915)
α_u_	0.0004	− 0.0003	− 1.9173***	0.0000	− 3.5718***	− 0.0927**	− 1.9741***	− 0.0951	− 1.2797***	− 1.8434***
	(7.7361)	(9.8548)	(1.0110)	(3.4547)	(0.5787)	(2.4699)	(1.6189)	(5.3823)	(0.4991)	(7.2499)
β_u_	0.0000	0.0000	− 0.7963***	0.0000	0.5627***	0.7256***	− 0.7207***	0.2630***	− 1.0380***	− 0.3952***
	(0.9048)	(0.6317)	(0.4677)	(1.0862)	(0.1756)	(0.7359)	(0.4717)	(1.1248)	(0.3663)	(0.7041)
AIC	− 0.0519	− 0.0395	− 0.9374	− 0.0479	**− 6.3606**	0.7178	− 0.5118	− 0.1049	− 3.0013	− 1.1128
5. Rotated Gumbel										
ὼ_L_	0.0002	0.0280***	− 1.5769***	− 0.0001	2.3363***	0.6275***	2.4658***	2.5354***	2.1542***	− 2.2195***
	(6.1091)	(0.5523)	(0.5678)	(2.7514)	(1.9088)	(4.1562)	(2.3172)	(2.8736)	(0.8240)	(0.8506)
α_L_	− 0.0002	− 0.0280***	1.7871***	0.0001	− 2.5249***	− 0.5698***	− 2.2817***	− 2.5336***	− 1.8818***	2.3294***
	(6.0324)	(0.5047)	(0.4707)	(2.6894)	(1.6329)	(3.9208)	(2.4898)	(2.6782)	(0.9127)	(0.7243)
β_L_	0.0000	0.0000	− 0.4939***	0.0000	0.8456***	− 0.0782***	− 0.5334***	− 0.0033	− 0.7455***	− 0.1615***
	(1.2696)	(0.4615)	(0.3205)	(0.5713)	(0.7417)	(0.7336)	(0.5830)	(0.8688)	(0.3395)	(0.4256)
AIC	− 0.0484	− 0.0385	− 2.5053	− 0.0325	− 1.5329	0.0889	− 1.1356	− 0.1988	− 1.1432	− 0.09052
6. Symmetrized JC										
ὼ_U_	− 20.6630***	− 17.7196***	− 20.4209***	− 20.8841***	− 16.6000***	− 16.9259***	− 21.1456***	− 20.4989***	− 20.5628***	− 19.9958***
	(1.0000)	(1.0000)	(1.0000)	(1.0000)	(97.2161)	(84.5235)	(1.0000)	(1.0000)	(1.0000)	(214.5680)
β_U_	0.0000	0.0000	0.0000	0.0000	− 0.5819	− 0.5202	0.0000	0.0000	0.0000	− 2.9315***
	(1.0000)	(1.0000)	(1.0000)	(1.0000)	(43.6792)	(48.0899)	(1.0000)	(1.0000)	(1.0000)	(76.4480)
α_U_	0.0000	0.0000	0.0000	0.0000	− 0.0152	0.0424	0.0000	0.0000	0.0000	− 0.0052
	(1.0000)	(1.0000)	(1.0000)	(1.0000)	(1.5146)	(4.0303)	(1.0000)	(1.0000)	(1.0000)	(1.0299)
ὼ_L_	− 18.0539***	− 17.0785***	− 18.0310***	− 18.1053***	− 20.1282***	− 16.9242***	− 17.9790***	− 18.2438***	− 18.2736***	− 16.6303
	(1.0000)	(1.0000)	(1.0000)	(1.0000)	(203.7393)	(58.2425)	(1.0000)	(1.0000)	(1.0000)	(86.5340)
β_L_	0.0000	0.0000	0.0000	0.0000	− 0.0174	− 0.1406	0.0000	0.0000	0.0000	− 0.5893
	(1.0000)	(1.0000)	(1.0000)	(1.0000)	(1.6406)	(13.0325)	(1.0000)	(1.0000)	(1.0000)	(30.7743)
α_L_	0.0000	0.0000	0.0000	0.0000	0.0001	− 0.0018	0.0000	0.0000	0.0000	− 0.0107
	(1.0000)	(1.0000)	(1.0000)	(1.0000)	(1.0001)	(1.0136)	(1.0000)	(1.0000)	(1.0000)	(1.1158)
AIC	28.9284	29.0128	26.3223	32.1495	12.2607	21.5866	30.3587	32.4637	34.1685	11.8308
7. Student's *t*										
Ψ_0_	0.0149***	− 0.0155***	− 0.0136***	− 0.0141***	0.0683***	0.0237***	0.0028***	− 0.0453***	− 0.0264***	0.0956***
	(0.0562)	(0.0376)	(0.0591)	(0.0588)	(0.0551)	(0.0518)	(0.0670)	(0.0665)	(0.0653)	(0.0690)
Ψ_1_	0.1202***	0.0727***	− 0.0562***	0.0774***	− 0.0917***	− 0.1197***	0.0732***	0.0266***	− 0.0704***	− 0.0831***
	(0.0774)	(0.0780)	(0.0812)	(0.0873)	(0.0815)	(0.0883)	(0.1235)	(0.0633)	(0.0653)	(0.1117)
Ψ_2_	− 1.3303***	− 0.0904***	− 1.5807***	− 1.5209***	− 0.6150***	− 1.0368***	− 1.7734***	− 2.0028***	− 2.0000***	− 1.4802***
	(0.9427)	(1.4809)	(0.5592)	(0.7299)	(1.2158)	(1.0548)	(0.3753)	(0.0015)	(0.0034)	(0.6809)
υ	5.0000***	5.0000***	5.0000***	5.0000***	5.0000***	5.0000***	5.0000***	5.0000***	5.0000***	5.0000***
	(0.4429)	(0.2625)	(2.9659)	(0.4070)	(0.9834)	(0.9220)	(1.1780)	(0.2902)	(0.4227)	(0.6335)
AIC	112.1165	121.4967	120.6067	132.3209	121.9518	120.7919	123.731	105.2099	127.6902	140.7409

Maximum Likelihood estimates for bivariate dynamic copulas are presented in the above table. Standard errors are presented in parenthesis. AIC values are adjusted for small− sample bias for each model with minimum AIC values (**in bold**) highlighting the best fitted copula. ***, ** and * denote 1%, 5% and 10% level of significance, respectively

5

## Abbreviations

MENA: Middle East and North African
C-QQR: Copula quantile-on-quantile method
QRA: Quantile regression analysis
GFC: Global financial crisis

## Footnotes

1 For more information on the CoVaR, see Mensi et al. (2017).

2 We have cited the study of Reboredo (2013b) as he puts forward the hedging relations between gold and oil using copula. Our approach also employs copula-based method.

3 Our C-QQR analysis is based on the student’s t copula. Gaussian copula is believed to have the convenience of its parameters which are correlation coefficients (Bouye and Salmon, 2009). However, student’s t copula also has the same convenience. Moreover, student’s t copula is better at capturing tail dependence, both upper and lower (Sim 2016).

4 For more details, please read Sim (2016) and Selmi et al. (2018).

5 To keep the presentation simple, the detailed outcomes of the dependence between gold and each of the rest of MENA countries will be available for interested readers upon request.

6 In the interest of brevity, we did not include Table 5 in the main text.

7 Normal copula suggests symmetric tail dependence while, Gumbel copula captures lower tail dependence, while rotated Gumbel considers upper tail dependence. In contrast, the Clayton copula assesses the upper tail dependence and rotated Clayton copula identifies lower tail dependence. For details, please see Mensi et al. (2017).

## References

[CR1] Akhtaruzzaman MD, Boubaker B, Lucey BM, Sensoy A (2021). Is gold a hedge or a safe-haven asset in the COVID–19 crisis?. Econ Model.

[CR2] Azour J, Zhu L (2020) Ensuring the benefits of capital flows to middle East. International monetary Fund. https://blogs.imf.org/2020/01/15/ensuring-the-benefits-of-capital-flows-in-the-middle-east/. Accessed 4 May 2020

[CR3] Batten JA, Ciner C, Lucey BM (2010). The macroeconomic determinants of volatility in precious metals markets. Resour Policy.

[CR4] Baur DG, Lucey BM (2010). Is gold a hedge or a safe haven? An analysis of stocks, bonds and gold. Financ Rev.

[CR5] Baur DG, McDermott TK (2010). Is gold a safe haven? International evidence. J Bank Finance.

[CR6] Beckers S, Soenen L (1984). Gold: more attractive to non-U.S than to U.S investors?. J Business Finance Accounting.

[CR7] Beckmann J, Czudaj R (2013). Gold as an inflation hedge in a time-varying coefficient framework. North Am J Econom Finance.

[CR8] Beckmann J, Berger T, Czudaj R (2015). Does gold act as a hedge or a safe haven for stocks? A smooth transition approach. Econ Model.

[CR9] Boubaker H, Cunado J, Gil-Alana L-A, Gupta R (2020). Global crises and gold as a safe haven: evidence from over seven and a half centuries of data. Physica A.

[CR10] Bouri E, Shahzad SJH, Roubaud D, Kristoufek L, Lucey B (2020). Bitcoin, gold, and commodities as safe havens for stocks: new insight through wavelet analysis. Quarter Rev Econom Finance.

[CR11] Bouyé E, Salmon M (2009). Dynamic copula quantile regressions and tail area dynamic dependence in Forex markets. European J Finance.

[CR12] Bredin D, Conlon T, Potì V (2015). Does gold glitter in the long-run? Gold as a hedge and safe haven across time and investment horizon. Int Rev Financ Anal.

[CR13] Chau F, Deesomsak R, Wang J (2014). Political uncertainty and stock market volatility in the Middle East and North African (MENA) countries. J Int Finan Markets Inst Money.

[CR14] Cheema MA, Faff RW, Szulczuk K (2020). The 2008 global financial crisis and COVID-19 pandemic: how safe are the safe haven assets?. Covid Economics.

[CR15] Chua J, Woodward RS (1982). Gold as an inflation hedge: a comparative study of six major industrial countries. J Bus Financ Acc.

[CR16] Chua JH, Sick G, Woodward RS (1990). Diversifying with gold stocks. Financ Anal J.

[CR17] Ciner C, Gurdgiev C, Lucey BM (2013). Hedges and safe havens: an examination of stocks, bonds, gold, oil and exchange rates. Int Rev Financ Anal.

[CR18] Cohen G, Qadan M (2010). Is gold still a shelter to fear. Am J Soc Manage Sci.

[CR19] Corbet S, (Greg) Hou, Y., Hu, Y., & Oxley, L.,  (2022). The influence of the COVID-19 pandemic on the hedging functionality of Chinese financial markets. Res Int Business Finance.

[CR20] Dar AB, Maitra D (2017). Is gold a weak or strong hedge and safe haven against stocks? Robust evidences from three major gold-consuming countries. Appl Econ.

[CR21] Embrechts P, McNeil AJ, Straumann D, Dempster M (2003). Correlation and dependency in risk management: properties and pitfalls. Risk management: value at risk and beyond.

[CR22] Girardi G, Ergün AT (2013). Systemic risk measurement: multivariate GARCH estimation of covar. J Bank Finance.

[CR100] Gürgün G, Ünalmış I (2014). Is gold a safe haven against equity market investment in emerging and developing countries?. Finance Res Lett.

[CR23] Hammoudeh S, Santos PA, Al-Hassan A (2013). Downside risk management and VaR-based optimal portfolios for precious metals, oil and stocks. North Am J Econom Finance.

[CR24] He Z, O'Connor F, Thijssen J (2018). Is gold a sometime safe haven or an always hedge for equity investors? A markov-switching CAPM approach for US and UK stock indices. Int Rev Financ Anal.

[CR25] Hillier D, Draper P, Faff R (2006). Do precious metals shine?. An Invest Perspect Financ Analysts J.

[CR26] Hood M, Malik F (2013). Is gold the best hedge and a safe haven under changing stock market volatility?. Rev Financ Econ.

[CR27] Jaffe JF (1989). Gold and gold stocks as investments for institutional portfolios. Financ Anal J.

[CR28] Ji Q, Zhang D, Zhao Y (2020). Searching for safe-haven assets during the COVID-19 pandemic. Int Rev Financ Analy.

[CR29] Joy M (2011). Gold and the US dollar: hedge or haven?. Financ Res Lett.

[CR30] Koenker R, & Hallock K (2000) Quantile regression: an introduction. Available at http://www.econ.uiuc.edu/;roger/ research/intro/intro.html&.

[CR31] Koenker R, Bassett B (1978). Regression quantiles. Econometrica.

[CR32] Kou G, Olgu Akdeniz Ö, Dinçer H, al.  (2021). Fintech investments in European banks: a hybrid IT2 fuzzy multidimensional decision-making approach. Financ Innov.

[CR33] Kroner KF, Ng VK (1998). Modeling asymmetric comovements of asset returns. Rev Financ Stud.

[CR34] Lucey BM, Tully E, Poti V (2003). International portfolio formation, skewness, and the role of gold. Front Finance Economics.

[CR35] Lucey BM, Li S (2015). What precious metals act as safe havens, and when? Some U.S. evidence. Appl Econom Lett.

[CR36] Maghyereh A-I, Awartani B, Tziogkidis P (2017). Volatility spillovers and cross-hedging between gold, oil and equities: evidence from the Gulf cooperation council countries. Energy Econom.

[CR37] Mensi W, Hammoudeh S, Reboredo JC, Nguyen DK (2015). Are Sharia stocks, gold and U.S. Treasury hedges and/or safe havens for the oil-based GCC markets?. Emerg Mark Rev.

[CR38] Mensi W, Hammoudeh S, Shahzad SHJ, Shahbaz M (2017). Modeling systemic risk and dependence structure between oil and stock markets using a variational mode decomposition-based copula method. J Bank Finance.

[CR39] Ming L, Shen Y, Yang S, Zhu S, Zhu H (2019). Does gold serve as a hedge for the stock market in China? Evidence from a time-frequency analysis. Emerg Mark Financ Trade.

[CR40] O'Connor FA, Lucey BM, Batten JA, Baur DG (2015). The financial economics of gold—a survey. Int Rev Financ Anal.

[CR41] Pandey V (2018). Volatility spillover from crude oil and gold to BRICS equity markets. J Econom Stud.

[CR42] Peng X (2019). Do precious metals act as hedges or safe havens for China's financial markets?. Finance Res Lett Forthcoming.

[CR43] Qureshi S, Rehman IU, Qureshi F (2018). Does gold act as a safe haven against exchange rate fluctuations? The case of Pakistan rupee. Journal of Policy Modeling.

[CR44] Raza N, Ibrahimy AI, Ali A, Ali S (2016). Gold and Islamic stocks: a hedge and safe haven comparison in time frequency domain for BRICS markets. J Develop Areas.

[CR45] Reboredo JC (2013). Is gold a safe haven or a hedge for the US dollar? Implications for risk management. J Bank Finance.

[CR46] Reboredo JC (2013). Is gold a hedge or safe haven against oil price movements?. Resour Policy.

[CR47] Robiyanto R, Nugroho BA, Handriani E, al.  (2020). Hedge effectiveness of put replication, gold, and oil on ASEAN-5 equities. Financ Innov.

[CR48] Salisu AA, Nadko UB, Oloko TF (2019). Assessing the inflation hedging of gold and palladium in OECD countries. Resour Policy.

[CR49] Selmi R, Mensi W, Hammoudeh S, Bouoiyour J (2018). Is Bitcoin a hedge, a safe haven or a diversifier for oil price movements? A comparison with gold. Energy Economics.

[CR50] Selmi R (2022) Inflation worries are back! does the transition to a carbon-neutral world will impact the role of commodities as an inflation hedge? Mimeo

[CR51] Shahzad SJH, Raza N, Shahbaz M, Ali A (2017). Dependence of stock markets with gold and bonds under bullish and bearish market states. Resour Policy.

[CR52] Shahzad SJH, Bouri E, Roubaud D, Kristoufek L, Lucey B (2019). Is bitcoin a better safe-haven investment than gold and commodities?. Int Rev Financ Anal.

[CR53] Shahzad., S.H.J., Mensi, W., Hammoudeh, S., Sohail, A., & Al-Yahyaee, K.H.  (2019). Does gold act as a hedge against different nuances of inflation? Evidence from quantile-on-quantile and causality-in- quantiles approaches. Resour Policy.

[CR54] Sim N (2016). Modeling the dependence structures of financial assets through the copula quantile-on-quantile approach. Int Rev Financ Anal.

[CR55] Sumner S, Johnson R, Soenen L (2010). Spillover effects among gold, stocks, and bonds. J Centrum Cathedra.

[CR56] Vigne SA, Lucey BM, O’Connor FA, Yarovaya L (2017). The financial economics of white precious metals—a survey. Int Rev Financ Analy.

[CR57] Wang K, Lee Y, Thi TN (2011). Time and place where gold acts as an inflation hedge: an application of long-run and short-run threshold model. Econ Model.

[CR58] Wen F, Xu L, Ouyang G, Kou G (2019). Retail investor attention and stock price crash risk: evidence from China. Int Rev Financ Analy.

